# Evolution of Dengue Virus Type 3 Genotype III in Venezuela: Diversification, Rates and Population Dynamics

**DOI:** 10.1186/1743-422X-7-329

**Published:** 2010-11-18

**Authors:** Alvaro Ramírez, Alvaro Fajardo, Zoila Moros, Marlene Gerder, Gerson Caraballo, Daria Camacho, Guillermo Comach, Victor Alarcón, Julio Zambrano, Rosa Hernández, Gonzalo Moratorio, Juan Cristina, Ferdinando Liprandi

**Affiliations:** 1 Laboratorio de Biología de Virus. Centro de Microbiología y Biología Celular. Instituto Venezolano de Investigaciones Científicas, Caracas, Venezuela; 2 Laboratorio de Virología Molecular. Centro de Investigaciones Nucleares. Facultad de Ciencias, Igua 4225, 11400 Montevideo, Uruguay; 3 Laboratorio Regional de Diagnóstico e Investigación del Dengue y Otras Enfermedades Virales - LARDIDEV, Maracay, Venezuela; 4 Departamento de Virología, Instituto Nacional de Higiene Rafael Rangel - INHRR, Ciudad Universitaria, Caracas, Venezuela

## Abstract

**Background:**

Dengue virus (DENV) is a member of the genus *Flavivirus *of the family *Flaviviridae*. DENV are comprised of four distinct serotypes (DENV-1 through DENV-4) and each serotype can be divided in different genotypes. Currently, there is a dramatic emergence of DENV-3 genotype III in Latin America. Nevertheless, we still have an incomplete understanding of the evolutionary forces underlying the evolution of this genotype in this region of the world. In order to gain insight into the degree of genetic variability, rates and patterns of evolution of this genotype in Venezuela and the South American region, phylogenetic analysis, based on a large number (*n *= 119) of envelope gene sequences from DENV-3 genotype III strains isolated in Venezuela from 2001 to 2008, were performed.

**Results:**

Phylogenetic analysis revealed an *in situ *evolution of DENV-3 genotype III following its introduction in the Latin American region, where three different genetic clusters (A to C) can be observed among the DENV-3 genotype III strains circulating in this region. Bayesian coalescent inference analyses revealed an evolutionary rate of 8.48 × 10^-4 ^substitutions/site/year (s/s/y) for strains of cluster A, composed entirely of strains isolated in Venezuela. Amino acid substitution at position 329 of domain III of the E protein (A→V) was found in almost all E proteins from Cluster A strains.

**Conclusions:**

A significant evolutionary change between DENV-3 genotype III strains that circulated in the initial years of the introduction in the continent and strains isolated in the Latin American region in recent years was observed. The presence of DENV-3 genotype III strains belonging to different clusters was observed in Venezuela, revealing several introduction events into this country. The evolutionary rate found for Cluster A strains circulating in Venezuela is similar to the others previously established for this genotype in other regions of the world. This suggests a lack of correlation among DENV genotype III substitution rate and ecological pattern of virus spread.

## Background

Dengue virus (DENV) is a member of the genus *Flavivirus *of the family *Flaviviridae*.

DENV are mosquito-borne flaviviruses with a single-stranded, nonsegmented, positive-sense RNA genome of approximately 11 kb in length [1]. Dengue viruses are comprised of four distinct serotypes (DENV-1 through DENV-4), which are transmitted to humans through the bites of two mosquito species: *Aedes aegypti *and *Aedes albopictus *[2].

DENV causes a wide range of diseases in humans, from the acute febrile illness dengue fever (DF) to life-threatening dengue hemorrhagic fever/dengue shock syndrome (DHF/DSS). Dengue has spread throughout tropical and subtropical regions worldwide over the past several decades, with an estimated 100 million infections and tens of millions of cases occurring annually [3]. Currently, there is a dramatic re-emergence of DENV in Latin America and an alarming increase of DF and DHF/DSS cases in this region [4].

Based on sequence analysis of the E/NS1 region, and using a cut-off of 6% divergence, each DENV serotype can be divided in different genotypes [5]. In the case of DENV-3, this serotype has been divided into four genotypes (I-IV) [6-8], sometimes including a genotype V [9].

Recent findings have demonstrated the emergence and global spread of DENV-3 genotype III [8]. The emergence of DHF in Sri Lanka in 1989 coincided with the appearance there of a new DENV-3, genotype III variant, which spread from the Indian subcontinent into Africa and Latin America [8]. Sri Lankan DENV-3 genotype III and associated American isolates have been linked to severe disease epidemics [10].

Phylogenetic analyses have elucidated the origins and forces underlying the molecular evolution of DENV in different geographic regions of the world [11]. Nevertheless, we still have an incomplete understanding of the dispersion and evolutionary history of DENV-3 genotype III in the South American region.

The objective of the present study was to gain insight into the degree of genetic variability, rates and patterns of evolution of this genotype in Venezuela and the South American region based on the analysis of a large number (*n *= 119) of envelope (E) gene sequences of DENV-3 genotype III strains isolated in Venezuela from 2001 to 2008.

## Results

### Genetic variability of DENV-3 genotype III circulating in Venezuela

In order to gain insight into the degree of genetic variability of DENV-3 genotype III strains circulating in Venezuela, 29 Venezuelan DENV-3 genotype III E gene sequences representing strains isolated between 2000 and 2007 in seven different Venezuelan geographic locations, were aligned with 58 sequences from DENV-3 genotype III E gene of DENV isolated in Latin America and 11 DENV-3 sequences from strains isolated elsewhere representing other DENV-3 genotypes (for strains included in these studies see Additional File 1, Table S1).

**Figure 1:**
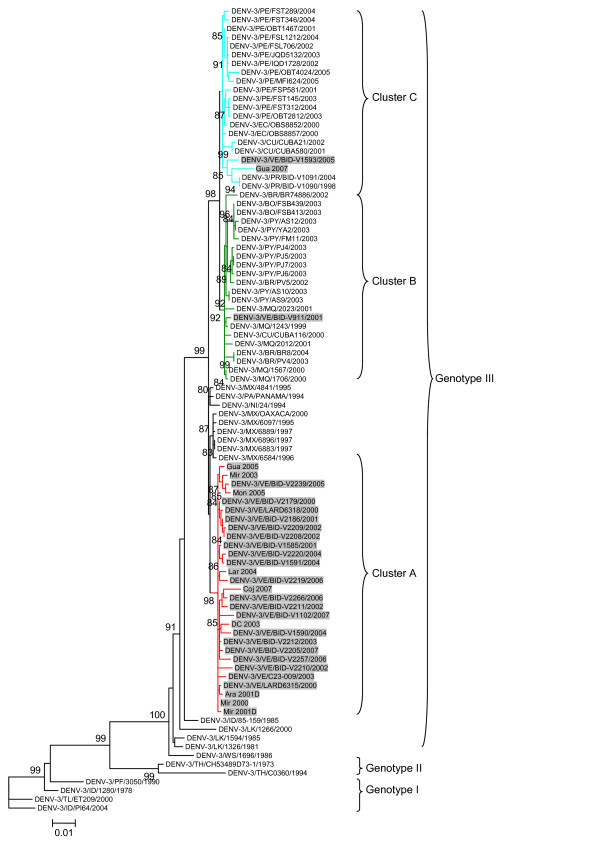
**Maximum likelihood phylogenetic tree analysis of DENV-3 genotype III strains isolated in Venezuela**. Strains in the trees are shown by the standardized terminology (which identifies their serotype, country, name and year of isolation) for strains previously described. Strains reported in these studies are shown by name. Venezuelan strains are shown highlighted in grey. Numbers at the branches show aLRT values. The scale bar indicates nucleotide substitutions per site. Cluster A branch is highlighted in red, clusters B and C branches are highlighted in green and light blue, respectively.

Once aligned, we first identified the optimal evolutionary model that best represent our sequence dataset (Akaike Information Criteria and Hierarchical Likelihood Ratio Test indicated that the GTR+Γ model fit the sequence data). Using this model, maximum likelihood phylogenetic trees were constructed and the robustness of each node of the tree was assessed by approximate Likelihood Ratio Test (aLRT). The results of these studies are shown in Figure 1.

All strains in the tree are assigned according to their genotype. Genotype III strains cluster together, strains belonging to other DENV-3 genotypes cluster separately (see Figure 1). These clusters are supported by very high values of aLRT. Inside genotype III cluster, three different clades can be observed for strains isolated in South America: one composed exclusively by strains isolated in Venezuela (Cluster A; Figure 1, bottom); one composed by strains isolated in Martinique, Brazil, Bolivia, Paraguay (Cluster B; Figure 1, middle); and one composed by strains isolated mainly in Ecuador and Peru (Cluster C; Figure 1 top). Clusters B and C also include strains isolated in the Caribbean region. Previous strains isolated in Nicaragua, Panama and Mexico from 1994 to 2000 were assigned to a different cluster inside genotype III (see Figure 1, middle). These strains circulated in the initial years after the introduction of DENV-3 genotype III in the continent, but show a great divergence with recent Latin American strains. This reflects a significant evolutionary change among strains isolated in the initial years and recent strains.

Interestingly, strains isolated in Venezuela are not only assigned to Cluster A, but also to Clusters B (strain DENV-3/VE/BID-V911/2001) and C (strains DENV-3/VE/BID-V1593/2005 and Gua2007), revealing at least three different introduction events of DENV-3 genotype III in that country (see Figure 1). The results of these studies also show the diversification of DENV-3 genotype III circulating in South America in three different genetic lineages (see Figure 1).

In order to confirm these findings, the same studies were done using a dataset containing all the 119 DENV-3 genotype III sequences isolated in Venezuela (for strains included in these studies see Additional File 1, Table S1). Using this dataset, we have found that apart from the three strains mentioned, the rest of the 119 DENV-3 genotype III strains isolated in Venezuela were assigned to Cluster A (Additional File 2, Fig. S2).

### Diversification of DENV-3 genotype III in the South American region

In order to gain insight into the diversification of DENV-3 genotype III in the South American region, full-length E protein amino acid sequences from strains isolated in Venezuela, representative of the three genotype III clusters observed, were aligned.

DENV-3 E protein resembles its homolog of DENV-2 in its dimeric structure and in the details of its protein folding [12]. Each monomer consists of three domains: domain I, an eight-stranded *β*-barrel, which organizes the structure; domain II, which contains 12 *β*-strands and two α-helices; and domain III, which is an IgG-like domain, with 10 *β*-strands. In solution and in the crystals, two monomers of E assemble head to tail to form a dimer [13].

**Figure 2:**
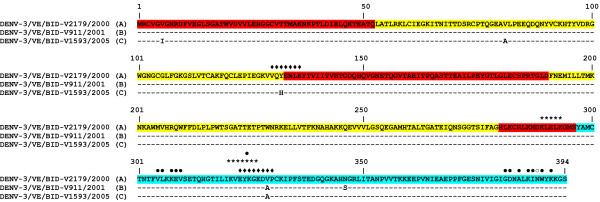
**Alignment of E protein ectodomain amino acid sequences from DENV-3 genotype III strains isolated in Venezuela**. Strains are listed by their names on the left side of the figure, and the cluster to which the strain is assigned is indicated between parentheses (see also Fig. 1). Only one strain of each cluster is shown (for detailed results of substitutions found in the rest of the Venezuelan strains see Additional File 2, Table S2). Identity to strain FJ639750 (DENV-3/VE/BID-V2179/2000) is indicated by a dash. Amino acid positions (relative to strain FJ639750) are shown by numbers at the top of the alignment. Sequences corresponding to E protein domains I, II and III are shown in red, yellow and light blue, respectively. Surface-exposed sequences previously identified on the dimeric DENV-3 protein are shown by a black diamond. Potential ELK/KLE-type and KELK/KLEK-type motifs are shown by a star. Asparagine at position 388 is shown by a white circle. Amino acid residues recently identified to be critical for neutralization by complex-reactive monoclonal antibodies (Mabs) on domain III of DENV-3 are indicated by a black circle [43].

As shown in Figure 2, Cluster A strains (composed entirely by genotype III strains isolated in Venezuela) present an amino acid substitution in position 329 (A→V), while strains from Clusters B and C share an Alanine at this position (see Figure 2, domain III). Interestingly, Cluster C strains present an amino acid substitution at position 132 (Y→H) with respect to Clusters A and B strains (see Figure 2, domain I), in a region previously described to be exposed at the surface of the protein [14]. Besides, the unique Venezuelan strain assigned to Cluster B present an amino acid substitution at position 346 (N→S) (see Figure 2, domain III). An asparagine (N) at position 388 was found in all strains included in these studies (see Figure 2). Previous studies in DENV-2 have shown that an N at this position appears to be associated to increased incidences of hemorrhagic fever (see Figure 2, domain III) [15].

These studies were repeated using all the 119 sequences of DENV-3 genotype III strains isolated in Venezuela. The same conclusions were obtained using this dataset (for detailed results of substitutions found in these strains in nucleotide and/or amino acids sequences, see Additional File 3, Table S2).

### Mapping of amino acid substitutions found in DENV-3 genotype III of strains circulating in the South American region

**Figure 3:**
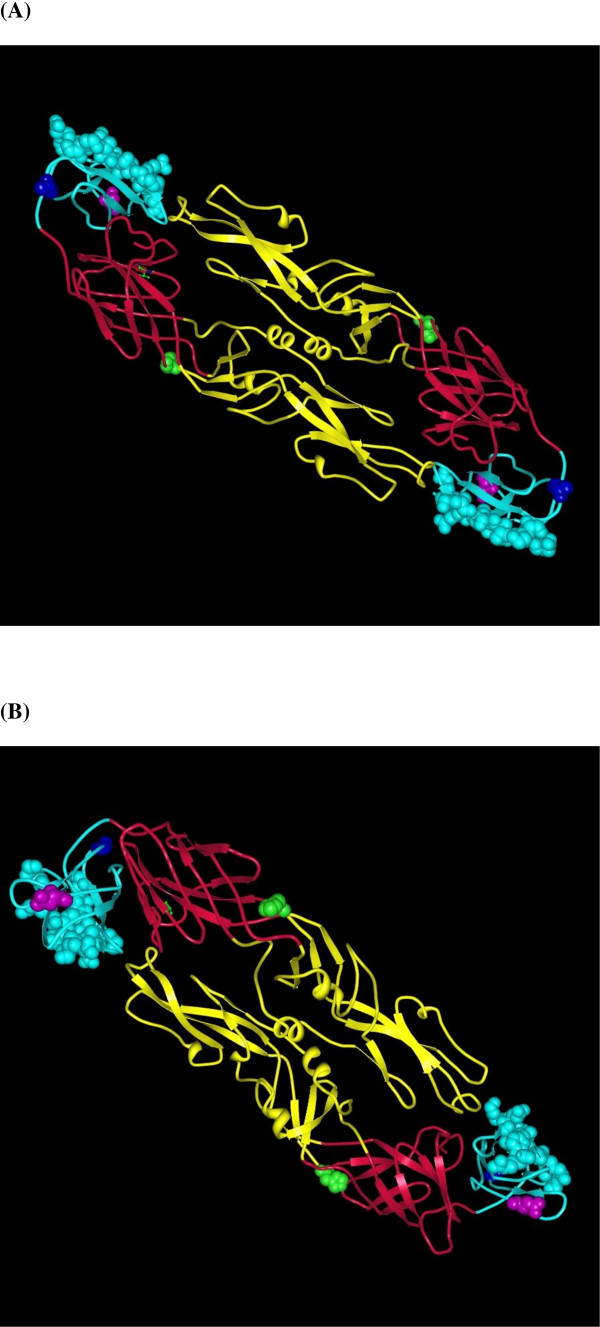
**Structure of the E protein dimer of DENV-3**. E protein domains I through III are indicated in red, yellow and light blue, respectively. Residues recently identified to be critical for neutralization [43] are shown in space-filling representation. Substitution at position 329 (A→V), found in cluster A strains, is shown in blue in space-filling representation. Substitution at position 346 (N→S), found in a Venezuelan Cluster B strain is shown in magenta in space-filling representation. Amino acid substitution at position 132 (Y→H), present in Cluster B with respect to Clusters A and C strains is shown in green in space-filling representation. Two views of the protein dimer, rotated over the z-axis, are shown in A and B.

In order to map the amino acid substitutions found on the E protein structure of DENV-3 genotype III strains of the three clusters identified in this study, we employed the PDB ProteinWorkshop 3.6 [16], using as a reference the E protein structure of DENV-3 strain DENV-3/TH/CH53489D73-1/1973 [12]. The results of these studies are shown in Figure 3.

Amino acid substitution at position 329 of domain III, found in Cluster A strains, is situated in previously identified surface-exposed amino acids in DENV-3 E protein [12,13,17]. The amino acid at position 132 of domain II is also exposed on the surface of the E protein (see Figure 3) [14].

### Bayesian coalescent analysis of DENV-3 genotype III Cluster A strains

In order to gain insight into the evolutionary rate and mode of evolution of DENV-3 genotype III circulating in Venezuela, we used a Bayesian Markov chain Monte Carlo (MCMC) approach as implemented in the BEAST package [18], to analyze E gene sequences of DENV-3 genotype III of strains included in Cluster A (see Figure 1). The results shown in Table 1 are the outcome of the analysis for 20 million steps of the MCMC, using the GTR+Γ model, a relaxed clock [19] and the expansion population growth model [20].

**Table 1:** Bayesian coalescent inference of Cluster A DENV-3 genotype III strains isolated in Venezuela.

Group*^a^*	Parameter	Value*^b^*	HPD*^c^*	ESS*^d^*
Cluster A	Log likelihood	-2727,7	-2738,8 to -2717,1	4586
	Posterior	-2798,5	-2816,8 to -2780,0	1123
	Prior	-70,76	-86,50 to -56,03	685
	Mean Rate*^e^*	8,48 × 10^-4 ^	5,62 × 10^-4 ^to 1,15 × 10^-3^	1451
	Root age (yr)	8,96	7,32 to 11,19	1013
	MRCA*^f^*	1998		

*^a ^*See Figure 1 and Supplementary Material Table 1 for strains included in this analysis.

*^b ^*In all cases, the main values are shown. *^c ^*HPD, high probability density values.

*^d ^*ESS, effective sample size. *^e ^*Mean rate is expressed in substitution/site/year.

*^f ^*MRCA, year of the most common recent ancestor.

As shown in Table 1, our results suggest that Cluster A, entirely composed by strains circulating in Venezuela, evolved from ancestors that existed around 1998. When the GTR+Γ model is used, a mean rate of 8.48 × 10^-4 ^nucleotide substitution per site per year was obtained for Cluster A strains (Table 1). This rate is similar to the ones obtained in similar studies for DENV-2 genotype III and DENV-4 genotype II (8.0 × 10^-4 ^and 8.3 × 10^-4 ^substitutions/site/year, respectively) circulating in the Americas [21]. This rate is also roughly similar to the others previously estimated for American DENV-3 genotype III strains (8,2 × 10^-4 ^and 1.03 × 10^-3 ^substitutions/site/year) [22,33].

## Discussion

After an absence of 17 years from the Latin American region, DENV-3 re-emerged in Central America in 1994 [23], and continue to expand into South America [8,17,24-32]. Previous studies have shown that this emerging DENV-3 is a genotype III variant of Asiatic origin [8,24-26,33]. Interestingly, the phylogenetic analysis presented in these studies reveal an *in situ *evolution of DENV-3 genotype III following its introduction in the Latin American region, where three different genetic clusters can be observed in DENV-3 genotype III strains circulating in the South American region (Figure 1). In addition, we observed a significant evolutionary change between DENV-3 genotype III strains that circulated in the initial years of the introduction in the continent (1994-2000) and strains isolated in the Latin American region in recent years (see Figure 1).

In this study, the evolution of DENV-3 genotype III in Venezuela was extensively analyzed. DENV-3 cases from Venezuela were first reported in the central region of the country [26]. Previous studies have proposed that a characteristic of dengue in Venezuela is that the outbreaks were first reported in neighboring countries, specifically in Central America and the Caribbean Islands, and then the epidemic spread northwards to Mexico and southern United States and southwards into South America. Therefore, the introduction of DENV-3 strains into Venezuela is more likely to have occurred as the result of the spread of strains circulating in Central America or the Caribbean islands and not to direct introduction or importation of Asiatic strains [26]. The phylogenetically closest strain to the earliest (year 2000) DENV-3 Venezuelan isolates is in fact an 1999 isolate from the geographically close Aruba island. This is in agreement with the results of this study, since strains isolated in Venezuela have been found in all genetic clusters of DENV-3 genotype III reported in this work (see Figure 1). Moreover, the presence of DENV-3 genotype III strains belonging to different clusters was observed in Venezuela in different years (2001, 2005 and 2007, see Figure 1). This reveals that several introduction events of DENV-3 genotype III strains take place in this country. Since the traveling history of the patients from which these isolates were obtained is not known, we cannot determine if these three isolates correspond simply to imported cases or form part of the circulation of minor variants that remain undetectable due to a low number of isolates available.

Nevertheless, the predominant type of DENV-3 strains circulating in Venezuelan belongs to cluster A (Figure 1). This cluster include strains isolated in Aragua State along several years (2000, 2001, 2002, 2003, 2004, 2006, 2007 and 2008), the rest of the strains having been isolated in several different geographic locations of Venezuela (Miranda, Monagas, Guarico, Lara and Cojedes States, and the Distrito Federal). Previous studies suggested that DENV-3 is evolving at a rate of 9.0 × 10^-4 ^substitutions/site/year (s/s/y) [34]. Very recent studies using much larger datasets revealed a similar rate (8.9 × 10^-4 ^s/s/y) [33]. This is in agreement with the results found in this study for DENV-3 genotype III cluster A strains entirely composed of strains isolated in Venezuela (8.48 × 10^-4 ^s/s/y, see Figure 1 and Table 1). Evolutionary rates for DENV-3 genotype III isolated elsewhere revealed roughly similar figures (11.6 × 10^-4 ^s/s/y [34], 10.3 × 10^-4 ^s/s/y [22] and 8.2 × 10^-4 ^s/s/y [33]. The differences between these estimations are probably due to the different number of sequences used in the studies, although lay within the confidence intervals of the estimations.

It has been previously suggested that the ecological conditions for DENV dissemination may alter the viral evolutionary rate among dengue lineages [34]. Nevertheless, the results of this study revealed that the main evolutionary rate found for DENV-3 genotype III Cluster A strains circulating in Venezuela (8.48 × 10^-4 ^s/s/y) is similar to others determined in different regions of the world, as well as for other serotypes. This suggests a lack of correlation among DENV genotype III substitution rate and ecological pattern of virus spread, in agreement with recent results [33].

The results of these studies suggest that Cluster A Venezuelan strains evolved from ancestors that existed around 1998 (1996-2000) (see Table 1). This result is consistent with very recent studies on DENV migration that suggests DENV-3 genotype III was introduced into the Americas through Mexico where this genotype was first isolated in 1995 [33], and a rapid spread to other countries in the region.

Amino acid substitution at position 329 of domain III, found in E proteins from Cluster A strains isolated in Venezuela, is situated in previously identified surface-exposed amino acids in DENV-3 E protein [12,13] (see Figure 3). Substitutions at this position have also been found in DENV-3 genotype III strains isolated in Ecuador and Peru [17]. This alanine (Ala)-to-valine (Val) substitution implies a change of a hydrophobic amino acid by another hydrophobic but aliphatic amino acid. While most amino acids contain only one non-hydrogen substituent attached to their C-beta carbon, Val contains two. This means that there is a lot more bulkiness near the protein backbone. Whether this may permit the virus to escape immune recognition or neutralization remains to be established.

## Conclusions

A significant evolutionary change between DENV-3 genotype III strains that circulated in the initial years of the introduction in the continent and strains isolated in the Latin American region in recent years was observed. The presence of DENV-3 genotype III strains belonging to different clusters was observed in several years. This fact reveals that several introduction events of DENV-3 genotype III strains take place in this country. The main evolutionary rate found for Cluster A strains circulating in Venezuela is similar to others previously established for this genotype in other regions of the world, as well as for other serotypes. This fact is in agreement with recent studies that suggest a lack of correlation among DENV genotype III substitution rate and ecological pattern of virus spread. Although a high degree of genetic variation has been observed among the three different clusters of DENV-3 genotype III strains circulating in the Latin American region, the E protein of these strains is relatively well conserved among all clusters.

More studies will be needed to characterize all DENV-3 genotype III clusters circulating in the Latin American region. This will permit to design appropriate anti-viral strategies against DENV infection.

## Methods

### Viruses

The new sequences of the E gene reported in this study are from Venezuelan strains of DENV-3, originally isolated in the "Department of Virology" from the "Instituto Nacional de Higiene Rafael Rangel - INHRR, Caracas, Venezuela, and from the "Laboratorio Regional de Diagnóstico e Investigación del Dengue y Otras Enfermedades Virales - LARDIDEV, Maracay, Venezuela. Acute-phase sera of patients identified as infected with DENV-3 using an established RT-PCR Multiplex protocol [35], were used to infect monolayers of the Aedes albopictus cell line C6/36. After growth for 7 days at 32°C, virus-infected supernatants were collected, clarified by centrifugation and stored at -70°C until use.

### RNA extraction and PCR amplification

Viral RNA was extracted from 280 μl supernatant medium of virus-infected cells using the QIAamp^® ^Viral RNA System, according to the manufacturer's protocol (Qiagen^®^, Chatsworth, CA, USA). Viral RNA was reverse transcribed to cDNA first strand in a 50-μl reaction final volume with Superscript II reverse transcriptase system (Invitrogen) and pd(N)6 random primers (Invitrogen, USA) or a specific primer (Additional File 4, Table S3). Reverse transcription was allowed to proceed at 42°C for 90 min followed by reverse transcriptase inactivation at 70°C for 15 min. cDNA amplification was performed with synthetic primers listed in Supplementary Material Table 1. Both sense and antisense primers were used to amplify a fragment containing the E coding region on the viral RNA. The reaction mixture contained, in a total volume of 50 μL, 2 to 10 μL of the cDNA, 1 mmol/L of MgSO_4_, 0.3 mmol/L of each dNTP, 0.2 μmol/L of each sense an antisense primers, and 2.5 U of platinum^® ^pfx DNA polymerase (Invitrogen). The amplification was carried out in a thermal cycler (Eppendorff) as follows: 35 cycles at 94°C for 30 seconds, 60°C for 1.5 min, and 68°C for 2.5 min, followed by a final incubation at 68°C for 5 minutes. Each PCR was run with positive and negative controls and the fragments were separated by 1% agarose gel electrophoresis, stained with 1 μg/ml ethidium bromide, and detected under ultraviolet light.

### Amplicon purification and sequencing reactions

Amplicons were purified using QIAquick PCR Purification Kit from QIAGEN, according to instructions from the manufacturers. The sequence reaction was carried out using the Big Dye DNA sequencing kit (Perkin-Elmer) on a 373 DNA sequencer apparatus (Perkin-Elmer). Alternatively, sequences were obtained from purified amplicons through a commercial sequencing service (Macrogene Inc, Seoul, Korea). Both strands of the PCR product were sequenced in order to avoid discrepancies. The E gene nucleotide sequences obtained from the DENV-3 Venezuelan strains were deposited in the GenBank Database under accession numbers [GenBank:HM348812] through [GenBank:HM348831], (see also Additional File 1, Table S1). Previously reported E gene sequences from DENV-3 isolated in Venezuela were obtained by means of the Virus Variation Database at the National Center for Biotechnology Information (NCBI) [36] (for strains names and accession numbers see Additional File 1, Table S1).

### Sequence Datasets

Different datasets were constructed to perform phylogenetic analyses. One included all DENV-3 genotype III E gene sequences isolated in Venezuela (*n *= 119) between 2000 and 2008, as well as 58 sequences from DENV-3 genotype III E gene of DENV isolated in Latin America and 11 DENV-3 sequences from strains isolated elsewhere representing other DENV-3 genotypes. We created another dataset that includes 29 selected Venezuelan DENV-3 genotype III E gene sequences representing strains isolated in seven different Venezuelan geographic locations between 2000 and 2007 and the same other strains, isolated elsewhere, included in the former dataset (for strain names and accession numbers, see Additional File 1, Table S1). For the selection of the 29 sequences of the latter dataset, we considered several variables in order to avoid biases in our results, including avoiding the presence of duplicates.

### Phylogenetic analysis

Sequences were aligned using the MUSCLE program [37]. Once aligned, we first tested whether a recombination event occurred on any of the sequences used in these studies. We used two approaches implemented in the SimPlot Program [38]: 1) a sliding window analysis of distances and 2) the bootscanning [39]. No recombinant strains were found in the dataset (not shown).

The program Modelgenerator [40] was used to identify the optimal evolutionary model (Akaike Information Criteria and Hierarchical Likelihood Ratio Test indicated that the GTR+Γ model fit the sequence data). Using this model, maximum likelihood trees were constructed using software from the PhyML program [41] (for details about the ML parameters, see Additional File 5, Table S4). As a measure of the robustness of each node, we employed an approximate Likelihood Ratio Test (aLRT), that assesses that the branch being studied provides a significant likelihood gain, in comparison with the null hypothesis that involves collapsing that branch but leaving the rest of the tree topology identical [42]. In order to gain insight into the evolutionary rate and mode of evolution of DENV-3 genotype III strains circulating in Venezuela, we used a Bayesian Markov chain Monte Carlo (MCMC) approach as implemented in the BEAST package v.1.4.8 [18], to analyze E gene sequences of DENV-3 genotype III of strains included in cluster A (Figure 1) isolated between 2000 and 2007. Using the GTR+Γ model and 20 million steps of MCMC, different population dynamic models were tested (constant population size, exponential population growth, expansion population growth, logistic population growth and Bayesian Skyline). Statistical uncertainty in the data was reflected by the 95% highest probability density (HPD) values. Results were examined using the TRACER v1.4 program [43] from the BEAST package. Convergence was assessed with ESS (effective sample size) values after a burn-in of 2 million steps. Models were compared by calculating the Bayes Factor (BF) [44] from the posterior output of each of the models using TRACER v1.4 program [42] as explained in BEAST website http://beast.bio.ed.ac.uk/Model_comparison. A log BF (natural log units) > 2.3 indicates strong evidence against the null model. The expansion population growth model was the best fit to the data.

### Mapping of amino acid substitutions in DENV-3 E protein 3D structure

In order to map amino acid substitutions found in Venezuelan DENV-3 genotype III E protein, crystallography data of the E protein of the strain DENV-3/TH/CH53489D73-1/1973 [12] was imported from Protein Data Bank [PDB:1uzg]. Visualization was done using PDB ProteinWorkshop 3.6 [16].

## Competing interests

The authors declare that they have no competing interests.

## Authors' contributions

JC and FL conceived of the study, and participated in its design and coordination. AR and AF have made substantial contributions to the design of the study, acquisition of data, have performed phylogenetic analysis and contributed to the interpretation of the data. ZM, MG, GC, DC, GC, VA, JZ and RH have made substantial and fundamental contributions to obtain the DENV strains described in this work, field work, culture of strains and have been involved in revising the manuscript critically for important intellectual content. GM participated in the phylogenetic analysis and contributed to the discussion of the results found. JC participated in the phylogenetic analysis and wrote the paper. FL helped to draft the manuscript and made substantial and fundamental contributions to the interpretation and discussion of the results found in this work. All authors read and approved the final manuscript.

## Supplementary Material

Additional file 1 **Origins of the DENV-3 strains**. Table describing name, accession numbers, year of isolation and country of isolation of the strains enrolled in these studies. Click here for file

Additional file 2 **Maximum likelihood phylogenetic tree analysis of DENV-3 strains isolated in the Latin American region**. Figure showing a phylogenetic tree analysis of DENV-3 strains isolated in the Latin American region. Click here for file

Additional file 3 **Analysis of nucleotide and amino acid substitutions found in E protein from Venezuelan DENV-3 genotype III strains**. Table showing nucleotide and amino acid substitutions found in E protein from Venezuelan DENV-3 genotype III strains. Click here for file

Additional file 4 **Primers used for specific amplification and sequencing of DENV-3 envelope (E) protein coding region**. Table of primers for amplification and sequencing of DENV-3 envelope (E) protein coding region. Click here for file

Additional file 5 **Statistics of the maximum likelihood analyses**. Table of parameters for maximum likelihood analysis. Click here for file
