# Supplementary material for: Evolution of Dengue Virus Type 3 Genotype III in Venezuela: Diversification, Rates and Population Dynamics
Source: Virol J. 2010 Nov 18;7:329. doi: 10.1186/1743-422X-7-329 (PMC2998486; doi:10.1186/1743-422X-7-329)
Supplement: Additional file 1 — Origins of the DENV-3 strains. Table describing name, accession numbers, year of isolation and country of isolation of the strains enrolled in these studies. [file 1743-422X-7-329-S1.DOC]

Additional File 1. Table S1. Origins of the DENV-3 strains*^a^*.

___________________________________________________________________________

Name Year of Country of Accession number

Isolation Isolation

___________________________________________________________________________

Aruba 1999 1999 Aruba HM348812

Mir 2000 2000 Miranda, Venezuela HM348813

Ara 2001 2001 Aragua, Venezuela HM348814

Ara 2001B 2001 Aragua, Venezuela HM348815

Ara 2001C 2001 Aragua, Venezuela HM348816

Ara 2001D 2001 Aragua, Venezuela HM348817

DC 2001A 2001 Caracas, Venezuela HM348818

DC 2001B 2001 Caracas, Venezuela HM348819

DC 2001C 2001 Caracas, Venezuela HM348820

Mir 2001A 2001 Miranda, Venezuela HM348821

Mir 2001B 2001 Miranda, Venezuela HM348822

Mir 2001C 2001 Miranda, Venezuela HM348823

Mir 2001D 2001 Miranda, Venezuela HM348824

Mir 2003 2003 Miranda, Venezuela HM348825

DC 2003 2003 Caracas, Venezuela HM348826

Lar 2004 2004 Lara, Venezuela HM348827

Mon 2005 2005 Monagas, Venezuela HM348828

Gua 2005 2005 Guárico, Venezuela HM348829

Coj 2007 2007 Cojedes, Venezuela HM348830

Gua 2007 2007 Guárico, Venezuela HM348831

Additional File 1. Table S1. Origins of the DENV-3 genotype III strains*^a^*. (Cont.).

___________________________________________________________________________

Name Year of Country of Accession number

Isolation Isolation

___________________________________________________________________________

DENV-3/VE/BID-V2174/2000 2000 Venezuela FJ639746

DENV-3/VE/BID-V2175/2000 2000 Venezuela FJ639747

DENV-3/VE/BID-V2178/2000 2000 Venezuela FJ639749

DENV-3/VE/BID-V2179/2000 2000 Venezuela FJ639750

DENV-3/VE/LARD5990/2000 2000 Venezuela AY146764

DENV-3/VE/LARD6007/2000 2000 Venezuela AY146765

DENV-3/VE/LARD6812/2000 2000 Venezuela AY146766

DENV-3/VE/LARD6315/2000 2000 Venezuela AY146767

DENV-3/VE/LARD6318/2000 2000 Venezuela AY146768

DENV-3/VE/LARD6397/2000 2000 Venezuela AY146769

DENV-3/VE/LARD6411/2000 2000 Venezuela AY146770

DENV-3/VE/LARD6456/2000 2000 Venezuela AY146771

DENV-3/VE/C02-003/2001 2001 Venezuela DQ367720

DENV-3/VE/C09-006/2001 2001 Venezuela DQ371245

DENV-3/VE/BID-V904/2001 2001 Venezuela EU482612

DENV-3/VE/BID-V906/2001 2001 Venezuela EU482613

DENV-3/VE/BID-V913/2001 2001 Venezuela EU482614

DENV-3/VE/BID-V1113/2001 2001 Venezuela EU529684

DENV-3/VE/BID-V1116/2001 2001 Venezuela EU529685

DENV-3/VE/BID-V1117/2001 2001 Venezuela EU529686

DENV-3/VE/BID-V1118/2001 2001 Venezuela EU529687

DENV-3/VE/BID-V903/2001 2001 Venezuela EU529688

Additional File 1. Table S1. Origins of the DENV-3 genotype III strains*^a^*. (Cont.).

___________________________________________________________________________

Name Year of Country of Accession number

Isolation Isolation

___________________________________________________________________________

DENV-3/VE/BID-V907/2001 2001 Venezuela EU529689

DENV-3/VE/BID-V908/2001 2001 Venezuela EU529690

DENV-3/VE/BID-V911/2001 2001 Venezuela EU529691

DENV-3/VE/BID-V1115/2001 2001 Venezuela EU569688

DENV-3/VE/BID-V912/2001 2001 Venezuela EU569689

DENV-3/VE/BID-V915/2001 2001 Venezuela EU569690

DENV-3/VE/BID-V916/2001 2001 Venezuela EU569691

DENV-3/VE/BID-V905/2001 2001 Venezuela EU660420

DENV-3/VE/BID-V1114/2001 2001 Venezuela FJ182015

DENV-3/VE/BID-V1585/2001 2001 Venezuela FJ373303

DENV-3/VE/LARD6666/2001 2001 Venezuela AY146772

DENV-3/VE/LARD6667/2001 2001 Venezuela AY146773

DENV-3/VE/LARD6668/2001 2001 Venezuela AY146774

DENV-3/VE/LARD6722/2001 2001 Venezuela AY146775

DENV-3/VE/LARD7110/2001 2001 Venezuela AY146776

DENV-3/VE/LARD7812/2001 2001 Venezuela AY146777

DENV-3/VE/LARD7984/2001 2001 Venezuela AY146778

DENV-3/VE/BID-V2180/2001 2001 Venezuela FJ639751

DENV-3/VE/BID-V2181/2001 2001 Venezuela FJ639752

DENV-3/VE/BID-V2182/2001 2001 Venezuela FJ639753

DENV-3/VE/BID-V2183/2001 2001 Venezuela FJ639754

DENV-3/VE/BID-V2184/2001 2001 Venezuela FJ639755

DENV-3/VE/BID-V2185/2001 2001 Venezuela FJ639756

Additional File1. Table S1. Origins of the DENV-3 genotype III strains*^a^*. (Cont.).

___________________________________________________________________________

Name Year of Country of Accession number

Isolation Isolation

___________________________________________________________________________

DENV-3/VE/BID-V2187/2001 2001 Venezuela FJ639757

DENV-3/VE/BID-V2188/2001 2001 Venezuela FJ639758

DENV-3/VE/BID-V2189/2001 2001 Venezuela FJ639759

DENV-3/VE/BID-V2190/2001 2001 Venezuela FJ639760

DENV-3/VE/BID-V2191/2001 2001 Venezuela FJ639761

DENV-3/VE/BID-V2192/2001 2001 Venezuela FJ639762

DENV-3/VE/BID-V2193/2001 2001 Venezuela FJ639763

DENV-3/VE/BID-V2195/2001 2001 Venezuela FJ639765

DENV-3/VE/BID-V2196/2001 2001 Venezuela FJ639766

DENV-3/VE/BID-V2197/2001 2001 Venezuela FJ639767

DENV-3/VE/BID-V2198/2001 2001 Venezuela FJ639768

DENV-3/VE/BID-V2199/2001 2001 Venezuela FJ639769

DENV-3/VE/BID-V2203/2001 2001 Venezuela FJ639770

DENV-3/VE/BID-V2204/2001 2001 Venezuela FJ639771

DENV-3/VE/BID-V2207/2001 2001 Venezuela FJ639774

DENV-3/VE/BID-V2186/2001 2001 Venezuela FJ744700

DENV-3/VE/BID-V2208/2002 2002 Venezuela FJ639775

DENV-3/VE/BID-V2209/2002 2002 Venezuela FJ639776

DENV-3/VE/BID-V2210/2002 2002 Venezuela FJ639777

DENV-3/VE/BID-V2211/2002 2002 Venezuela FJ639778

DENV-3/VE/C23-009/2003 2003 Venezuela DQ367721

DENV-3/VE/C29-008/2003 2003 Venezuela DQ367722

DENV-3/VE/BID-V2212/2003 2003 Venezuela FJ639779

Additional File 1. Table S1. Origins of the DENV-3 genotype III strains*^a^*. (Cont.).

___________________________________________________________________________

Name Year of Country of Accession number

Isolation Isolation

___________________________________________________________________________

DENV-3/VE/BID-V2213/2003 2003 Venezuela FJ639780

DENV-3/VE/BID-V2214/2003 2003 Venezuela FJ639781

DENV-3/VE/BID-V2215/2003 2003 Venezuela FJ639782

DENV-3/VE/BID-V2217/2003 2003 Venezuela FJ639784

DENV-3/VE/BID-V2218/2003 2003 Venezuela FJ639785

DENV-3/VE/BID-V1591/2004 2004 Venezuela EU854291

DENV-3/VE/BID-V1590/2004 2004 Venezuela FJ373304

DENV-3/VE/BID-V2220/2004 2004 Venezuela FJ639787

DENV-3/VE/BID-V2222/2004 2004 Venezuela FJ639789

DENV-3/VE/BID-V2223/2004 2004 Venezuela FJ639790

DENV-3/VE/BID-V2224/2004 2004 Venezuela FJ639791

DENV-3/VE/BID-V2225/2004 2004 Venezuela FJ639792

DENV-3/VE/BID-V2226/2004 2004 Venezuela FJ639793

DENV-3/VE/BID-V2228/2004 2004 Venezuela FJ639795

DENV-3/VE/BID-V2231/2004 2004 Venezuela FJ639798

DENV-3/VE/BID-V2232/2004 2004 Venezuela FJ639799

DENV-3/VE/BID-V2233/2004 2004 Venezuela FJ639800

DENV-3/VE/BID-V2234/2004 2004 Venezuela FJ639801

DENV-3/VE/BID-V1593/2005 2005 Venezuela EU854292

DENV-3/VE/BID-V2239/2005 2005 Venezuela FJ639803

DENV-3/VE/BID-V2240/2005 2005 Venezuela FJ639804

DENV-3/VE/BID-V2242/2005 2005 Venezuela FJ639805

DENV-3/VE/BID-V2244/2005 2005 Venezuela FJ639807

Additional File 1. Table S1. Origins of the DENV-3 genotype III strains*^a^*. (Cont.).

___________________________________________________________________________

Name Year of Country of Accession number

Isolation Isolation

___________________________________________________________________________

DENV-3/VE/BID-V2247/2005 2005 Venezuela FJ639810

DENV-3/VE/BID-V2256/2005 2005 Venezuela FJ639816

DENV-3/VE/BID-V2257/2006 2006 Venezuela FJ639817

DENV-3/VE/BID-V2266/2006 2006 Venezuela FJ639825

DENV-3/VE/BID-V2219/2006 2006 Venezuela FJ639786

DENV-3/VE/BID-V2205/2007 2007 Venezuela FJ639772

DENV-3/VE/BID-V1102/2007 2007 Venezuela EU529683

DENV-3/VE/BID-V2267/2008 2008 Venezuela FJ639826

DENV-3/VE/BID-V2268/2008 2008 Venezuela FJ639827

DENV-3/BO/FSB413/2003 2003 Bolivia DQ177886

DENV-3/BO/FSB439/2003 2003 Bolivia DQ177887

DENV-3/BR/BR74886/2002 2002 Brazil AY679147

DENV-3/BR/PV5/2002 2002 Brazil DQ118875

DENV-3/BR/PV4/2003 2003 Brazil DQ118 874

DENV-3/BR/BR8/2004 2004 Brazil DQ118864

DENV-3/CU/CUBA116/2000 2000 Cuba AY702032

DENV-3/CU/CUBA580/2001 2001 Cuba AY702030

DENV-3/CU/CUBA21/2002 2002 Cuba AY702031

DENV-3/EC/OBS8852/2000 2000 Ecuador DQ177898

DENV-3/EC/OBS8857/2000 2000 Ecuador DQ177899

DENV-3/EC/EC8241/2000 2000 Ecuador FM246468

DENV-3/EC/EC5080/2001 2001 Ecuador FM246467

DENV-3/EC/EC9110/2003 2003 Ecuador FM246470

Additional File 1. Table S1. Origins of the DENV-3 genotype III strains*^a^*. (Cont.).

___________________________________________________________________________

Name Year of Country of Accession number

Isolation Isolation

___________________________________________________________________________

DENV-3/EC/EC8801/2004 2004 Ecuador FM246469

DENV-3/EC/EC15082/2004 2004 Ecuador FM246472

DENV-3/EC/EC9266/2005 2005 Ecuador FM246473

DENV-3/EC/EC9233/2005 2005 Ecuador FM246471

DENV-3/EC/EC4860/2007 2007 Ecuador FM246466

DENV-3/MQ/1243/1999 1999 Martinique AY099337

DENV-3/MQ/1567/2000 2000 Martinique AY099338

DENV-3/MQ/1706/2000 2000 Martinique AY099339

DENV-3/MQ/2012/2001 2001 Martinique AY099340

DENV-3/MQ/2023/2001 2001 Martinique AY099341

DENV-3/MX/6097/1995 1995 Mexico AY146763

DENV-3/MX/4841/1995 1995 Mexico DQ341202

DENV-3/MX/6584/1996 1996 Mexico DQ341203

DENV-3/MX/6883/1997 1997 Mexico DQ341204

DENV-3/MX/6889/1997 1997 Mexico DQ341205

DENV-3/MX/6896/1997 1997 Mexico DQ341206

DENV-3/MX/OAXACA/2000 2000 Mexico DQ341207

DENV-3/NI/24/1994 1994 Nicaragua AY702033

DENV-3/PA/PANAMA/1994 1994 Panama DQ341209

DENV-3/PY/AS10/2003 2003 Paraguay DQ118883

DENV-3/PY/AS12/2003 2003 Paraguay DQ118884

DENV-3/PY/AS9/2003 2003 Paraguay DQ118885

DENV-3/PY/FM11/2003 2003 Paraguay DQ118886

Additional File 1. Table S1. Origins of the DENV-3 genotype III strains*^a^*. (Cont.).

___________________________________________________________________________

Name Year of Country of Accession number

Isolation Isolation

___________________________________________________________________________

DENV-3/PY/PJ4/2003 2003 Paraguay DQ118887

DENV-3/PY/PJ5/2003 2003 Paraguay DQ118888

DENV-3/PY/PJ6/2003 2003 Paraguay DQ118889

DENV-3/PY/PJ7/2003 2003 Paraguay DQ118890

DENV-3/PY/YA2/2003 2003 Paraguay DQ118891

DENV-3/PE/FSP581/2001 2001 Peru DQ177890

DENV-3/PE/OBT1467/2001 2001 Peru DQ177900

DENV-3/PE/FSL706/2002 2002 Peru DQ177889

DENV-3/PE/IQD1728/2002 2002 Peru DQ177895

DENV-3/PE/OBT2812/2003 2003 Peru DQ177901

DENV-3/PE/FST145/2003 2003 Peru DQ177891

DENV-3/PE/JQD5132/2003 2003 Peru DQ177896

DENV-3/PE/FST289/2004 2004 Peru DQ177892

DENV-3/PE/FST312/2004 2004 Peru DQ177893

DENV-3/PE/FST346/2004 2004 Peru DQ177894

DENV-3/PE/FSL1212/2004 2004 Peru DQ177888

DENV-3/PE/MFI624/2005 2005 Peru DQ177897

DENV-3/PE/OBT4024/2005 2005 Peru DQ177902

DENV-3/PR/BID-V1090/1998 1998 Puerto Rico EU529703

DENV-3/PR/BID-V1091/2004 2004 Puerto Rico EU529704

____________________________________________________________________________

*^a^*Strains highlighted in grey were enrolled in the Bayesian coalescent analysis.
