# Supplementary material for: Evolution of Dengue Virus Type 3 Genotype III in Venezuela: Diversification, Rates and Population Dynamics
Source: Virol J. 2010 Nov 18;7:329. doi: 10.1186/1743-422X-7-329 (PMC2998486; doi:10.1186/1743-422X-7-329)
Supplement: Additional file 2 — Maximum likelihood phylogenetic tree analysis of DENV-3 strains isolated in the Latin American region. Figure showing a phylogenetic tree analysis of DENV-3 strains isolated in the Latin American region. [file 1743-422X-7-329-S2.DOC]

**DENV-3/EC/EC8801/2004**

**DENV-3/EC/EC15082/2004**

**DENV-3/PE/FST346/2004**

**DENV-3/EC/EC9110/2003**

**DENV-3/PE/IQD1728/2002**

**DENV-3/EC/EC4860/2007**

**DENV-3/PE/MFI624/2005**

**DENV-3/PE/OBT4024/2005**

**DENV-3/PE/FSL706/2002**

**DENV-3/PE/FSL1212/2004**

**DENV-3/PE/JQD5132/2003**

**DENV-3/EC/EC9233/2005**

**DENV-3/PE/OBT1467/2001**

**DENV-3/PE/FSP581/2001**

**DENV-3/PE/FST145/2003**

**DENV-3/PE/FST312/2004**

**DENV-3/PE/OBT2812/2003**

**DENV-3/EC/OBS8852/2000**

**DENV-3/EC/EC8241/2000**

**DENV-3/PE/FST289/2004**

**DENV-3/EC/OBS8857/2000**

**DENV-3/EC/EC9266/2005**

**DENV-3/CU/CUBA21/2002**

**DENV-3/CU/CUBA580/2001**

**DENV-3/EC/EC5080/2001**

**DENV-3/PR/BID-V1090/1998**

**DENV-3/PR/BID-V1091/2004**

**DENV-3/VE/BID-V1593/2005**

**Gua 2007**

**DENV-3/BR/BR74886/2002**

**DENV-3/MQ/1243/1999**

**DENV-3/MQ/2023/2001**

**DENV-3/PY/AS9/2003**

**DENV-3/PY/AS10/2003**

**DENV-3/BR/PV5/2002**

**DENV-3/PY/PJ6/2003**

**DENV-3/PY/PJ7/2003**

**DENV-3/PY/PJ5/2003**

**DENV-3/PY/PJ4/2003**

**DENV-3/PY/FM11/2003**

**DENV-3/PY/YA2/2003**

**DENV-3/PY/AS12/2003**

**DENV-3/BO/FSB413/2003**

**DENV-3/BO/FSB439/2003**

**DENV-3/MQ/1706/2000**

**DENV-3/MQ/1567/2000**

**DENV-3/MQ/2012/2001**

**DENV-3/VE/BID-V911/2001**

**DENV-3/CU/CUBA116/2000**

**DENV-3/BR/PV4/2003**

**DENV-3/BR/BR8/2004**

**DENV-3/PA/PANAMA/1994**

**DENV-3/NI/24/1994**

**DENV-3/MX/4841/1995**

**DENV-3/MX/OAXACA/2000**

**DENV-3/MX/6097/1995**

**DENV-3/MX/6889/1997**

**DENV-3/MX/6896/1997**

**DENV-3/MX/6883/1997**

**DENV-3/MX/6584/1996**

**Aruba 1999**

**DENV-3/VE/BID-V1114/2001**

**DENV-3/VE/BID-V905/2001**

**DENV-3/VE/BID-V912/2001**

**DENV-3/VE/BID-V2219/2006**

**DENV-3/VE/BID-V2175/2000**

**DENV-3/VE/BID-V913/2001**

**DENV-3/VE/LARD6667/2001**

**DENV-3/VE/BID-V2196/2001**

**DENV-3/VE/BID-V2179/2000**

**DENV-3/VE/LARD6397/2000**

**DENV-3/VE/BID-V916/2001**

**DENV-3/VE/BID-V2209/2002**

**DENV-3/VE/BID-V2208/2002**

**DENV-3/VE/BID-V2186/2001**

**DENV-3/VE/LARD6668/2001**

**DENV-3/VE/LARD6411/2000**

**DENV-3/VE/LARD6318/2000**

**DENV-3/VE/LARD7984/2001**

**DENV-3/VE/BID-V2180/2001**

**DENV-3/VE/BID-V2181/2001**

**DENV-3/VE/BID-V2182/2001**

**DENV-3/VE/BID-V2183/2001**

**DENV-3/VE/BID-V2184/2001**

**DENV-3/VE/LARD7812/2001**

**DC 2001C 2**

**DENV-3/VE/BID-V2231/2004**

**Lar 2004**

**DENV-3/VE/BID-V2224/2004**

**DENV-3/VE/BID-V2233/2004**

**DENV-3/VE/C29-008/2003**

**DENV-3/VE/BID-V2223/2004**

**DENV-3/VE/BID-V2242/2005**

**DENV-3/VE/BID-V2222/2004**

**Mir 2003**

**DENV-3/VE/BID-V2239/2005**

**Mon 2005**

**Gua 2005**

**DENV-3/VE/BID-V2187/2001**

**DENV-3/VE/BID-V1118/2001**

**DENV-3/VE/BID-V2257/2006**

**DENV-3/VE/BID-V2232/2004**

**DENV-3/VE/BID-V2213/2003**

**DENV-3/VE/BID-V2207/2001**

**DENV-3/VE/BID-V1113/2001**

**DENV-3/VE/BID-V2195/2001**

**DENV-3/VE/BID-V2240/2005**

**DENV-3/VE/BID-V2217/2003**

**DENV-3/VE/BID-V2215/2003**

**DENV-3/VE/BID-V2205/2007**

**DENV-3/VE/BID-V2189/2001**

**DENV-3/VE/BID-V2212/2003**

**DENV-3/VE/BID-V1117/2001**

**DENV-3/VE/BID-V907/2001**

**DENV-3/VE/BID-V906/2001**

**Ara 2001 B**

**DENV-3/VE/BID-V2199/2001**

**DENV-3/VE/BID-V2247/2005**

**DENV-3/VE/BID-V2268/2008**

**DENV-3/VE/BID-V2267/2008**

**DENV-3/VE/BID-V1102/2007**

**DENV-3/VE/BID-V915/2001**

**DENV-3/VE/BID-V2256/2005**

**DENV-3/VE/BID-V2226/2004**

**DC 2003**

**DENV-3/VE/BID-V2210/2002**

**DENV-3/VE/BID-V2198/2001**

**DENV-3/VE/BID-V2192/2001**

**DENV-3/VE/BID-V1590/2004**

**DENV-3/VE/BID-V2266/2006**

**Coj 2007**

**DENV-3/VE/BID-V2211/2002**

**DENV-3/VE/C23-009/2003**

**DENV-3/VE/BID-V2185/2001**

**DENV-3/VE/BID-V2174/2000**

**DENV-3/VE/BID-V2225/2004**

**DENV-3/VE/BID-V2178/2000**

**DENV-3/VE/BID-V2203/2001**

**DENV-3/VE/LARD6456/2000**

**DENV-3/VE/BID-V2190/2001**

**DENV-3/VE/LARD6007/2000**

**DENV-3/VE/LARD7110/2001**

**DENV-3/VE/BID-V1585/2001**

**DENV-3/VE/BID-V2220/2004**

**DENV-3/VE/BID-V2218/2003**

**DENV-3/VE/BID-V2228/2004**

**DENV-3/VE/BID-V2244/2005**

**DENV-3/VE/BID-V2234/2004**

**DENV-3/VE/BID-V2214/2003**

**DENV-3/VE/BID-V1591/2004**

**DENV-3/VE/BID-V2193/2001**

**DENV-3/VE/BID-V1115/2001**

**DENV-3/VE/LARD6666/2001**

**DENV-3/VE/BID-V2197/2001**

**DENV-3/VE/BID-V908/2001**

**DENV-3/VE/BID-V903/2001**

**DENV-3/VE/BID-V1116/2001**

**DENV-3/VE/BID-V904/2001**

**DENV-3/VE/LARD5990/2000**

**DENV-3/VE/BID-V2191/2001**

**DENV-3/VE/C02-003/2001**

**DENV-3/VE/C09-006/2001**

**Mir 2000**

**Ara 2001C**

**DENV-3/VE/LARD6812/2000**

**DENV-3/VE/LARD6722/2001**

**DENV-3/VE/BID-V2204/2001**

**DENV-3/VE/BID-V2188/2001**

**DENV-3/VE/LARD6315/2000**

**Ara 2001D**

**DC 2001A 2**

**DC 2001B 2**

**Mir 2001A**

**Mir 2001B**

**Mir 2001C**

**Ara 2001**

**Mir 2001D**

**DENV-3/ID/85-159/1985**

**DENV-3/LK/1266/2000**

**DENV-3/WS/1696/1986**

**DENV-3/LK/1326/1981**

**DENV-3/LK/1594/1985**

**DENV-3/TH/CH53489D73-1/1973**

**DENV-3/TH/C0360/1994**

**DENV-3/PF/3050/1990**

**DENV-3/ID/1280/1978**

**DENV-3/TL/ET209/2000**

**DENV-3/ID/PI64/2004**

82

88

85

87

82

85

93

88

95

92

92

83

92

96

97

83

85

89

80

90

91

84

83

87

84

81

81

95

86

86

97

84

88

97

90

91

88

85

89

81

94

98

99

83

84

99

99

98

99

0.01

Genotype I

Genotype II

Cluster C

Cluster B

Cluster A

Genotype III
