# Supplementary material for: Evolution of Dengue Virus Type 3 Genotype III in Venezuela: Diversification, Rates and Population Dynamics
Source: Virol J. 2010 Nov 18;7:329. doi: 10.1186/1743-422X-7-329 (PMC2998486; doi:10.1186/1743-422X-7-329)
Supplement: Additional file 3 — Analysis of nucleotide and amino acid substitutions found in E protein from Venezuelan DENV-3 genotype III strains. Table showing nucleotide and amino acid substitutions found in E protein from Venezuelan DENV-3 genotype III strains. [file 1743-422X-7-329-S3.DOC]

**Additional File 3. Table S2. Analysis of nucleotide and amino acid substitutions found in E protein from Venezuelan DENV-3 genotype III strains*^a^*.**

|  |  |  |  |
| --- | --- | --- | --- |
| Strain | 242*^b^* (81) | 394 (132) | 986 (329) |
| EU854292 (2005) | C (Ala) | C (His) | C (Ala) |
| EU529691 (2001) | T (Val) | T (Tyr) | C (Ala) |
| Gua2007 (2007) | C (Ala) | T (Tyr) | T (Val) |
| Mir2000 (2000) | T (Val) | T (Tyr) | T (Val) |
| FJ639746 (2000) | T (Val) | T (Tyr) | T (Val) |
| FJ639747 (2000) | T (Val) | T (Tyr) | T (Val) |
| FJ639749 (2000) | T (Val) | T (Tyr) | T (Val) |
| FJ639750 (2000) | T (Val) | T (Tyr) | T (Val) |
| AY146764 (2000) | T (Val) | T (Tyr) | T (Val) |
| AY146765 (2000) | T (Val) | T (Tyr) | T (Val) |
| AY146766 (2000) | T (Val) | T (Tyr) | T (Val) |
| AY146767 (2000) | T (Val) | T (Tyr) | T (Val) |
| AY146768 (2000) | T (Val) | T (Tyr) | T (Val) |
| AY146769 (2000) | T (Val) | T (Tyr) | T (Val) |
| AY146770 (2000) | T (Val) | T (Tyr) | T (Val) |
| AY146771 (2000) | T (Val) | T (Tyr) | T (Val) |
| ARA2001 (2001) | T (Val) | T (Tyr) | T (Val) |
| ARA2001B (2001) | T (Val) | T (Tyr) | T (Val) |
| ARA2001C (2001) | T (Val) | T (Tyr) | T (Val) |
| ARA2001D (2001) | T (Val) | T (Tyr) | T (Val) |
| DC2001A (2001) | T (Val) | T (Tyr) | T (Val) |
| DC2001B (2001) | T (Val) | T (Tyr) | T (Val) |
| DC2001C (2001) | T (Val) | T (Tyr) | T (Val) |
| Mir2001A (2001) | T (Val) | T (Tyr) | T (Val) |
| Mir2001B (2001) | T (Val) | T (Tyr) | T (Val) |
| Mir2001C (2001) | T (Val) | T (Tyr) | T (Val) |
| Mir2001D (2001) | T (Val) | T (Tyr) | T (Val) |
| DQ367720 (2001) | T (Val) | T (Tyr) | T (Val) |
| DQ371245 (2001) | T (Val) | T (Tyr) | T (Val) |
| EU482612 (2001) | T (Val) | T (Tyr) | T (Val) |
| EU482613 (2001) | T (Val) | T (Tyr) | T (Val) |
| EU482614 (2001) | T (Val) | T (Tyr) | T (Val) |
| EU529684 (2001) | T (Val) | T (Tyr) | T (Val) |
| EU529685 (2001) | T (Val) | T (Tyr) | T (Val) |
| EU529686 (2001) | T (Val) | T (Tyr) | T (Val) |
| EU529687 (2001) | T (Val) | T (Tyr) | T (Val) |
| EU529688 (2001) | T (Val) | T (Tyr) | T (Val) |
| EU529689 (2001) | T (Val) | T (Tyr) | T (Val) |
| EU569688 (2001) | T (Val) | T (Tyr) | T (Val) |
| EU569689 (2001) | T (Val) | T (Tyr) | T (Val) |
| EU569690 (2001) | T (Val) | T (Tyr) | T (Val) |
| EU569691 (2001) | T (Val) | T (Tyr) | T (Val) |
| EU660420 (2001) | T (Val) | T (Tyr) | T (Val) |
| FJ182015 (2001) | T (Val) | T (Tyr) | T (Val) |
| FJ373303 (2001) | T (Val) | T (Tyr) | T (Val) |
| AY146772 (2001) | T (Val) | T (Tyr) | T (Val) |
| AY146773 (2001) | T (Val) | T (Tyr) | T (Val) |
| AY146774 (2001) | T (Val) | T (Tyr) | T (Val) |
| AY146775 (2001) | T (Val) | T (Tyr) | T (Val) |
| AY146776 (2001) | T (Val) | T (Tyr) | T (Val) |
| AY146777 (2001) | T (Val) | T (Tyr) | T (Val) |
| AY146778 (2001) | T (Val) | T (Tyr) | T (Val) |
| FJ639751 (2001) | T (Val) | T (Tyr) | T (Val) |
| FJ639752 (2001) | T (Val) | T (Tyr) | T (Val) |
| FJ639753 (2001) | T (Val) | T (Tyr) | T (Val) |
| FJ639754 (2001) | T (Val) | T (Tyr) | T (Val) |
| FJ639755 (2001) | T (Val) | T (Tyr) | T (Val) |
| FJ639756 (2001) | T (Val) | T (Tyr) | T (Val) |
| FJ639757 (2001) | T (Val) | T (Tyr) | T (Val) |
| FJ639758 (2001) | T (Val) | T (Tyr) | T (Val) |
| FJ639759 (2001) | T (Val) | T (Tyr) | T (Val) |
| FJ639760 (2001) | T (Val) | T (Tyr) | T (Val) |
| FJ639761 (2001) | T (Val) | T (Tyr) | T (Val) |
| FJ639762 (2001) | T (Val) | T (Tyr) | T (Val) |
| FJ639763 (2001) | T (Val) | T (Tyr) | T (Val) |
| FJ639765 (2001) | T (Val) | T (Tyr) | T (Val) |
| FJ639766 (2001) | T (Val) | T (Tyr) | T (Val) |
| FJ639767 (2001) | T (Val) | T (Tyr) | T (Val) |
| FJ639768 (2001) | T (Val) | T (Tyr) | T (Val) |
| FJ639769 (2001) | T (Val) | T (Tyr) | T (Val) |
| FJ639770 (2001) | T (Val) | T (Tyr) | T (Val) |
| FJ639771 (2001) | T (Val) | T (Tyr) | T (Val) |
| FJ639774 (2001) | T (Val) | T (Tyr) | T (Val) |
| FJ744700 (2001) | T (Val) | T (Tyr) | T (Val) |
| FJ639775 (2002) | T (Val) | T (Tyr) | T (Val) |
| FJ639776 (2002) | T (Val) | T (Tyr) | T (Val) |
| FJ639777 (2002) | T (Val) | T (Tyr) | T (Val) |
| FJ639778 (2002) | T (Val) | T (Tyr) | T (Val) |
| DC (2003) | T (Val) | T (Tyr) | T (Val) |
| Mir (2003) | T (Val) | T (Tyr) | T (Val) |
| DQ337721 (2003) | T (Val) | T (Tyr) | T (Val) |
| DQ337722 (2003) | T (Val) | T (Tyr) | T (Val) |
| FJ639779 (2003) | T (Val) | T (Tyr) | T (Val) |
| FJ639780 (2003) | T (Val) | T (Tyr) | T (Val) |
| FJ639781 (2003) | T (Val) | T (Tyr) | T (Val) |
| FJ639782 (2003) | T (Val) | T (Tyr) | T (Val) |
| FJ639784 (2003) | T (Val) | T (Tyr) | T (Val) |
| FJ639785 (2003) | T (Val) | T (Tyr) | T (Val) |
| Lar (2004) | T (Val) | T (Tyr) | T (Val) |
| EU854291 (2004) | T (Val) | T (Tyr) | T (Val) |
| FJ373304 (2004) | T (Val) | T (Tyr) | T (Val) |
| FJ639787 (2004) | T (Val) | T (Tyr) | T (Val) |
| FJ639789 (2004) | T (Val) | T (Tyr) | T (Val) |
| FJ639790 (2004) | T (Val) | T (Tyr) | T (Val) |
| FJ639791 (2004) | T (Val) | T (Tyr) | T (Val) |
| FJ639792 (2004) | T (Val) | T (Tyr) | T (Val) |
| FJ639793 (2004) | T (Val) | T (Tyr) | T (Val) |
| FJ639795 (2004) | T (Val) | T (Tyr) | T (Val) |
| FJ639798 (2004) | T (Val) | T (Tyr) | T (Val) |
| FJ639799 (2004) | T (Val) | T (Tyr) | T (Val) |
| FJ639800 (2004) | T (Val) | T (Tyr) | T (Val) |
| FJ639801 (2004) | T (Val) | T (Tyr) | T (Val) |
| Gua (2005) | T (Val) | T (Tyr) | T (Val) |
| Mon (2005) | T (Val) | T (Tyr) | T (Val) |
| FJ639803 (2005) | T (Val) | T (Tyr) | T (Val) |
| FJ639804 (2005) | T (Val) | T (Tyr) | T (Val) |
| FJ639805 (2005) | T (Val) | T (Tyr) | T (Val) |
| FJ639807 (2005) | T (Val) | T (Tyr) | T (Val) |
| FJ639810 (2005) | T (Val) | T (Tyr) | T (Val) |
| FJ639816 (2005) | T (Val) | T (Tyr) | T (Val) |
| FJ639817 (2006) | T (Val) | T (Tyr) | T (Val) |
| FJ639825 (2006) | T (Val) | T (Tyr) | T (Val) |
| FJ639786 (2006) | T (Val) | T (Tyr) | T (Val) |
| Coj (2007) | T (Val) | T (Tyr) | T (Val) |
| FJ639772 (2007) | T (Val) | T (Tyr) | C (Ala) |
| EU529683 (2007) | T (Val) | T (Tyr) | T (Val) |
| FJ639826 (2008) | T (Val) | T (Tyr) | T (Val) |
| FJ639827 (2008) | T (Val) | T (Tyr) | T (Val) |

*^a^* Strains are listed by their accession numbers for strains previously described, followed by their year of isolation. Strains reported in this work are shown by name (for accession numbers, see Supplementary Material Table 1). *^b^* Numbers indicate the nucleotide position in DENV-3 E gene, relative to strain [GenBank:EU854292] (Cluster B). The relative amino acid position, as well as the amino acid residue, is shown between parentheses.
