# Supplementary material for: Evolution of Dengue Virus Type 3 Genotype III in Venezuela: Diversification, Rates and Population Dynamics
Source: Virol J. 2010 Nov 18;7:329. doi: 10.1186/1743-422X-7-329 (PMC2998486; doi:10.1186/1743-422X-7-329)
Supplement: Additional file 4 — Primers used for specific amplification and sequencing of DENV-3 envelope (E) protein coding region. Table of primers for amplification and sequencing of DENV-3 envelope (E) protein coding region. [file 1743-422X-7-329-S4.DOC]

**Additional File4, Table S3. Primers used for specific amplification and sequencing of DENV-3 envelope (E) protein coding region.**

**_____________________________________________________________________________**

**Reaction Position Sequences (5`→3`)**

**_____________________________________________________________________________**

Reverse Transcription 2576 (antisense) CACTCCATTCTCCCCAAGCG

PCR (gen E) 796 (sense) CGAGAAGGTAGAGATGGGC

PCR (gen E) 2576 (antisense) CACTCCATTCTCCCCAAGCG

Sequencing (gen E) 796 (sense) CGAGAAGGTAGAGATGGGC

Sequencing (gen E) 1184 (antisense) GAGGAGCAGGACCAGAACTACGT

Sequencing (gen E) 1530 (antisense) TGAAGAACAAAGCATGGATGGT

Sequencing (gen E) 1944 (antisense) GAGGATGGACAAGGGAAAGC

Sequencing (gen E) 2576 (antisense) CACTCCATTCTCCCCAAGCG

______________________________________________________________________________
