# Supplementary material for: Evolution of Dengue Virus Type 3 Genotype III in Venezuela: Diversification, Rates and Population Dynamics
Source: Virol J. 2010 Nov 18;7:329. doi: 10.1186/1743-422X-7-329 (PMC2998486; doi:10.1186/1743-422X-7-329)
Supplement: Additional file 5 — Statistics of the maximum likelihood analyses. Table of parameters for maximum likelihood analysis. [file 1743-422X-7-329-S5.DOC]

**Additional File 5. Table S4. Statistics of the Maximum Likelihood analyses*.**

|  | A | B |
| --- | --- | --- |
| Model of nucleotides substitution | GTR | GTR |
| Number of taxa | 188 | 92 |
| Log-likelihood | -5.167 | -5.618 |
| Discrete gamma model | Yes | Yes |
| Number of categories | 4 | 4 |
| Gamma shape parameter | 1,804 | 3,354 |
| Proportion of invariant | 0,525 | 0,611 |
| Nucleotides frequencies |  |  |
| f(A) | 0,33369 | 0,31639 |
| f(C) | 0,20462 | 0,19496 |
| f(G) | 0,2631 | 0,27122 |
| f(T) | 0,19858 | 0,21743 |
| GTR relative rate parameters |  |  |
| A <-> C | 2,00251 | 3,87270 |
| A <-> G | 13,62382 | 23,60633 |
| A <-> T | 1,36501 | 3,46199 |
| C <-> G | 0,83282 | 1,43509 |
| C <-> T | 48,09178 | 78,37373 |
| G <-> T | 1 | 1 |

**Details of the Maximum Likelihood trees performed.** Two different Maximum Likelihood trees were constructed using two different datasets: one (A) containing all DENV-3 genotype III E gene sequences isolated in Venezuela (*n* = 119) between 2000 and 2008, as well as 58 sequences from DENV-3 genotype III E gene of DENV isolated in Latin America and 11 DENV-3 sequences from strains isolated elsewhere representing other DENV-3 genotypes. The other one (B) including 29 selected Venezuelan DENV-3 genotype III E gene sequences, isolated between 2000 and 2007, representing strains isolated in seven different Venezuelan geographic locations and the same other strains, isolated elsewhere, included in the former dataset.
